# Echocardiographic-Fluoroscopic Fusion Imaging Improves Interventionalists’ Learning Curve for Percutaneous Left Atrial Appendage Closure—Initial, Single-Center, Retrospective Observations

**DOI:** 10.3390/jcdd11030082

**Published:** 2024-02-29

**Authors:** Dominika Kanschik, Houtan Heidari, Kathrin Klein, Amin Polzin, Verena Veulemans, Jürgen Leick, Malte Kelm, Christian Jung, Tobias Zeus, Shazia Afzal

**Affiliations:** 1 Division of Cardiology, Pneumology and Vascular Medicine, University Hospital Düsseldorf, 40225 Düsseldorf, Germany; dominikaanna.kanschik@med.uni-duesseldorf.de (D.K.); houtan.heidari@med.uni-duesseldorf.de (H.H.); kathrin.klein@med.uni-duesseldorf.de (K.K.); amin.polzin@med.uni-duesseldorf.de (A.P.); verena.veulemans@med.uni-duesseldorf.de (V.V.); malte.kelm@med.uni-duesseldorf.de (M.K.); christian.jung@med.uni-duesseldorf.de (C.J.); 2 Heartcenter Trier, Krankenhaus der Barmherzigen Brüder, 54292 Trier, Germany; j.leick@bbtgruppe.de (J.L.); s.afzal@bbtgruppe.de (S.A.)

**Keywords:** structural heart diseases, left atrial appendage closure, fusion imaging

## Abstract

Due to the complex and variable anatomy of the left atrial appendage, percutaneous left atrial appendage closure (LAAC) can be challenging. In this study, we investigated the impact of fusion imaging (FI) on the LAAC learning curve of two interventionalists. The first interventionalist (IC 1) was initially trained without FI and continued his training with FI. The second interventionalist (IC 2) performed all procedures with FI. We compared the first 36 procedures without FI of IC 1 (group 1) with his next 36 interventions with FI (group 2). Furthermore, group 1 was compared to 36 procedures of IC 2 who directly started his training with FI (group 3). Group 1 demonstrated that the learning curve without FI has a flat course with weak correlations for fluoroscopy time, contrast volume, and procedure time, but not for dose area product. Group 2 with FI showed improvement with a steep course and strong correlations for all four parameters. In group 3, we also saw a steep progression with strong correlations. Furthermore, the mean measurements of the parameters in the groups with FI decreased significantly as an indicator of procedural efficacy. We demonstrated that FI may improve the learning curve of experienced and non-experienced ICs.

## 1. Introduction

Percutaneous left atrial appendage closure (LAAC) has become a commonly used alternative for stroke prevention worldwide in patients with non-valvular atrial fibrillation, who are not eligible for oral anticoagulation (class IIb recommendation) [1]. Large multicenter trials, such as PROTECT AF, PREVAIL, or PRAGUE-17, have already shown that LAAC provides comparable stroke prevention to vitamin K antagonists (VKAs) or novel oral anticoagulants (NOACs), with a reduction in complications such as bleeding or mortality [2,3]. Periprocedural imaging is essential when performing LAAC due to the considerable differences in size, shape, and relationship to neighboring structures such as the left pulmonary artery, left upper pulmonary vein, and circumflex artery [4]. Moreover, percutaneous interventions involve challenges such as navigation of the catheter and implantation of the device on a beating heart as well as the learning curve of the interventionalists [5]. Recent studies demonstrated that by improving aspects such as the design of the devices, periprocedural imaging, and the experience of cardiologists, the complication rate of peri-interventional life-threatening events such as pericardial effusion with cardiac tamponade, ischemic stroke, arrhythmias, bleeding, and increased 30-day mortality was significantly reduced [6]. Intraprocedural steps such as a transseptal puncture (TSP), optimal device sizing, and ultimately the implantation of the occluder device require great precision, because underestimation can lead to dislocation of the occluder device or peri-device leak, and oversizing may cause tamponade or embolization as well [7,8]. Thus, optimizing the interventionalist’s learning curve is of foremost importance as emphasized by current European and American expert consensus [9,10]. Previous studies have shown that the performance and safety of percutaneous LAAC have steadily improved with operator experience and that 30 procedures are required to reach proficiency and optimize clinical outcomes [11,12]. It is recommended to perform procedural imaging with transoesophageal echocardiography (TEE) or intracardiac echocardiography (ICE) [6,13]. Unfortunately, although TEE allows visualization of the soft tissues of the cardiac structures, there are limitations in the visualization of the catheter systems and devices used, therefore the simultaneous use of fluoroscopy is indispensable [14]. Through the application of fusion imaging (FI), the interventionalist no longer needs to mentally combine data from both imaging modalities, which can be very complex and requires good spatial imagination [15]. Instead, they are fused in real time and displayed on one screen and on the same image. Thus, guidance and navigation of catheters or devices while performing procedures can be facilitated [16]. We recently demonstrated the procedural advantages of FI for LAAC [17]: It reduces the procedure time, the time to transseptal puncture, and the periprocedural amount of contrast agent. Data about the impact of real-time echocardiography-fluoroscopy fusion imaging (FI) on the interventionalist’s learning curve during LAAC are lacking. Therefore, we aimed to evaluate its impact on an interventional cardiologist’s (IC) learning curve.

## 2. Materials and Methods

### 2.1. Study Design

**Figure 1:**
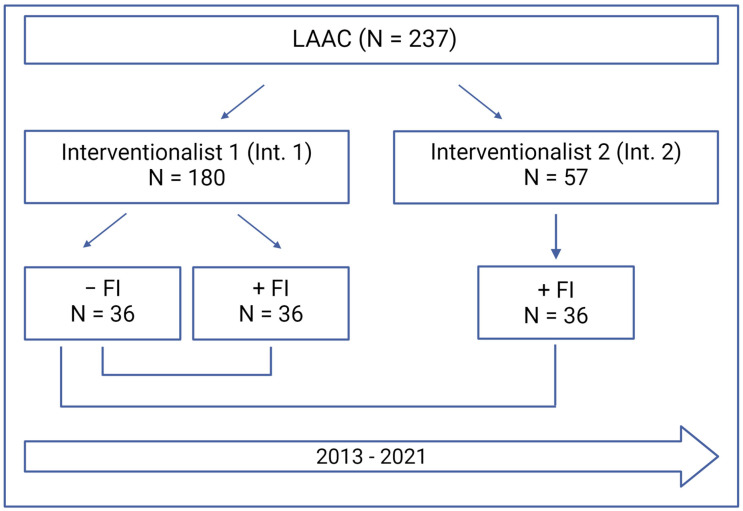
Study flowchart. LAAC—Left atrial appendage closure; FI—Fusion imaging.

We performed a retrospective single-center study and analyzed data of two interventional cardiologists (ICs), who performed 273 LAAC between 2011 and 2022 at our heart center (see Figure 1). The first interventionalist (IC 1) was experienced in both coronary and structural interventions (level of competence V defined by EAPCI [18]) and worked with FI (N = 180; +FI) and without FI (N = 36; −FI). The second interventionalist (IC 2) was also experienced (level of competence IV) but was a novice at LAAC. Furthermore, he performed all procedures with FI (N = 57) as the FI technique was already established when he started his training.

The first 36 procedures without FI by IC 1 (group 1) were compared with his next 36 interventions with FI (group 2). Furthermore, group 1 was analyzed with the first 36 procedures by IC 2, which were performed with FI right from the beginning (group 3). According to the Declaration of Helsinki, the study was approved by the local ethics committee (number 5272R) and registered at clinical trials (NCT02608008). Written informed consent was obtained from all patients.

### 2.2. Intervention

All procedures were performed minimally invasively under conscious sedation and local anesthesia in our catheterization laboratory according to a standardized protocol [19]. Only TEE and fluoroscopy were used for periprocedural guidance. Initially, TEE was used to exclude a possible left atrial appendage (LAA) thrombus and active endocarditis, which would be a contraindication to performing the intervention. A radial arterial catheter was inserted to monitor and measure hemodynamics. Under echocardiographic and fluoroscopic guidance, the transseptal puncture was performed posteriorly and inferiorly. After a successful transseptal puncture, the transseptal sheath and a pigtail catheter were advanced to the left atrium, and anticoagulation with heparin was started to prevent potential thrombus formation. During the examination, the activated clotting time was regularly monitored to achieve a target value of approximately 250 s. The sizing of the implanted device was based on the maximum Landing zone diameter according to the manufacturer’s instructions. After the implantation, we confirmed the final position of the device using TEE, tug-test, and injections of contrast medium.

### 2.3. Real-Time Echocardiography-Fluoroscopy Fusion (FI) Imaging during LAAC

**Figure 2:**
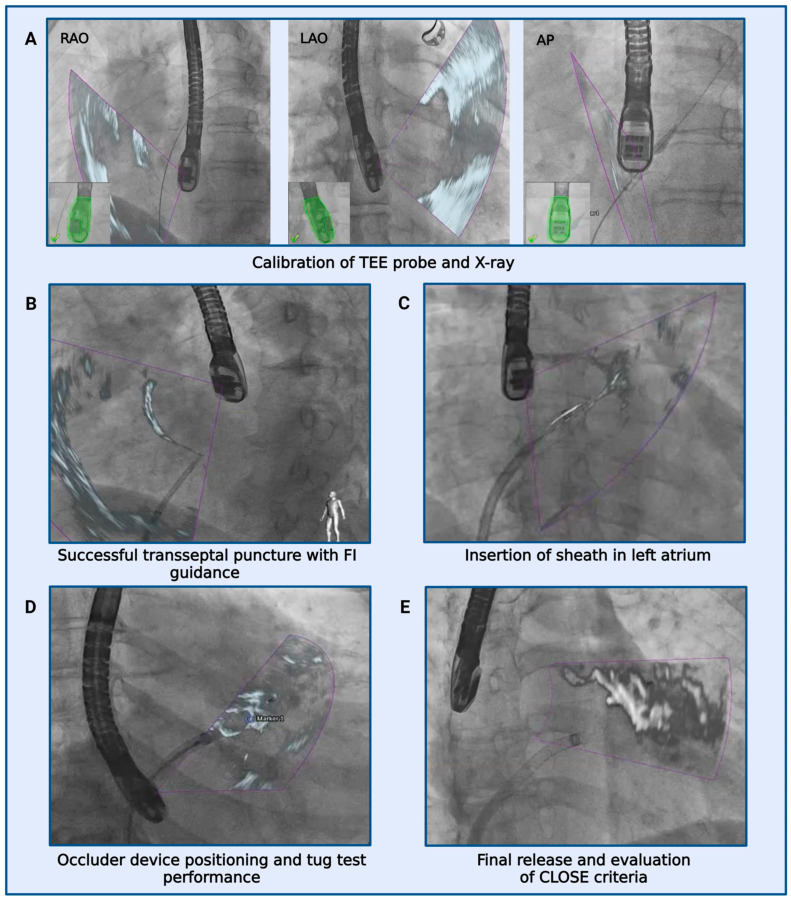
LAAC step by step under FI II guidance. (**A**): Green color confirmed correct positioning of the TEE probe. (**B**–**E**): Purple triangle depict real-time fluoroscopic and echocardiographic overlay.

FI has been recently introduced and aims to facilitate essential steps during structural heart disease interventions [20]. It is a sophisticated tool that allows a real-time two- and/or three-dimensional TEE imaging and fluoroscopic imaging overlay using the EchoNavigator^®^ System Release II (Philips Healthcare, Andover, MA, USA). Calibration of the TEE probe with fluoroscopy is achieved by an automatic tracking and localization of the TEE probe in relation to the C-arm angulation, based on fluoroscopic imaging data. The calibration was performed in Right Anterior Oblique (RAO) 30°/Cranial 20° und RAO 30°/Caudal 20° and within a few seconds. When the co-registration was successful, both the C-arm and TEE probes were detected, and an overlay of both imaging modalities was displayed in real time. Furthermore, static markers could be set to gain a better orientation in regions of interest such as the site of the TSP, coumadin ridge, and circumflex artery. Fluoroscopy is essential for catheter and device visualization whereas echocardiography provides spatial orientation. When the image is readjusted, these markers are automatically updated. With echocardiographic data continuously displayed within the fluoroscopic images, it was possible to accurately follow the anatomy of the soft tissue anatomy under continuous accordance with respiratory and cardiac cycle movements. The performance of LAAC is described step-by-step in Figure 2 and can be seen in Videos S1–S5.

### 2.4. Learning Curve and Procedural Parameters

To evaluate the learning curve, the changes in procedure parameters over time were considered and presented as correlations. The following procedural parameters were investigated: procedural success, procedure time (PT), contrast volume (CV), fluoroscopy time (FT), and dose area product (DAP). Outcome analysis included patient characteristics and complications according to the Munich consensus [21] such as pericardial effusion, bleeding, vascular complications, ischemic and hemorrhagic stroke, dislocation, and arrhythmia.

Successful performance of the procedure was defined as successful implantation of an occluder device. Total procedure time was defined as the time from local anesthesia to completion of TEE with documentation of a good result. Fluoroscopy time was expressed in minutes, whereas the area-dose product was expressed in cGy·cm^2^. To evaluate and assess the severity of bleeding symptoms we used the ISTH/SSC bleeding assessment tool [22]. All hemorrhages with a score of 4 points (blood transfusion, replacement therapy, or desmopressin) were considered. Arteriovenous fistula, pseudoaneurysm, arterial stenosis, or groin bleeding were all considered vascular complications.

### 2.5. Statistical Analysis

Statistical data analysis was performed using SPSS Statistics (version 27.0, SPSS Inc., IBM, Armonk, NY, USA). To prove the normal distribution of the data the Shapiro–Wilk test was performed. Categorical variables were expressed as absolute numbers with percentages. Continuous variables were expressed as medians with interquartile range (IQR) or mean with standard deviation (SD). For continuous variables, the Mann–Whitney U Test or Kruskal–Wallis were used. The learning curves were analyzed by correlation analysis by Spearman’s rho. Statistical significance was defined at *p*-Values < 0.05.

## 3. Results

### 3.1. Patient Characteristics

The mean age was between 74 and 78 years (75 ± 9 vs. 78 ± 6 vs. 75 ± 9, *p* = 0.222). There were no significant differences in patient characteristics between the three groups besides Has-Bled Score: 3 ± 1 vs. 4 ± 1 vs. 2 ± 1 (*p* < 0.001). The most common comorbidities included: arterial hypertension (88.9% vs. 91.7% vs. 94.4%, *p* = 0.695), hypercholesterolemia (83.3% vs. 77.8% vs. 72.2%, *p* = 0.526), and coronary artery disease (CAD) (80.6% vs. 61.1% vs. 55.6%, *p* = 0.064). Further details are displayed in Table 1.

### 3.2. Learning Curve Analyses

**Figure 3:**
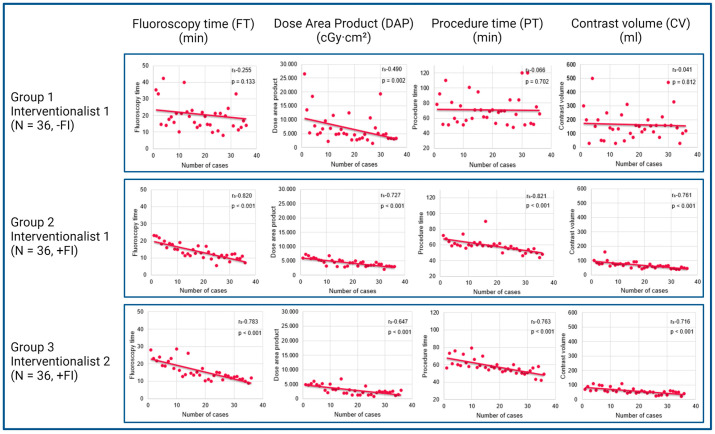
The course of the learning curve over time. In group 1 the learning curve was flat, and the correlations were weak except for DAP. In groups 2 and 3 the course was steep with strong correlations for all parameters. FI—Fusion imaging.

All procedures were performed successfully with the ACP/Amplatzer™ Amulet™ LAA Occluder (Abbott, Chicago, IL, USA). The courses of the learning curves over time are presented in Figure 3.

### 3.3. Learning Curve Analysis of Interventionalist 1

Group 1 (Interventionalist 1, without FI) reveals a flat course with weak correlations for fluoroscopy time (r_s_ = −0.255, *p* = 0.133), procedure time (r_s_ = −0.066, *p* = 0.702) and contrast volume (r_s_ = −0.041, *p* = 0.812). Only for the dose area product, the correlation was moderate (r_s_ = −0.490, *p* = 0.002).

Group 2 (Interventionalist 1, with FI) showed improvement with a steep course and strong correlations for all parameters: fluoroscopy time (r_s_ = −0.820, *p* < 0.001), dose area product (r_s_ = −0.727, *p* < 0.001), procedure time (r_s_ = −0.821, *p* < 0.001), and contrast volume (r_s_ = −0.761, *p* < 0.001).

### 3.4. Learning Curve Analysis of Interventionalist 2

The analysis of IC 2 (group 3) learning curve revealed a steep progression of the learning curve and strong correlations were shown as follows: fluoroscopy time (r_s_ = −0.783, *p* < 0.001), dose area product (r_s_ = −0.647, *p* = 0.001), procedure time (r_s_ = −0.763, *p* < 0.001), and contrast volume (r_s_ = −0.716, *p* = 0.001).

### 3.5. Analysis of FI’s Impact on Learning Curves

As the next step, we assessed the influence of FI on the learning curves by comparing the IC 1 learning curve with (group 1) and without FI (group 2). Furthermore, we evaluated the IC 1 and 2 learning curves (groups 2 and 3) with FI, and with the IC 1 curve without FI (group 1). Procedural parameters in the groups with FI improved significantly compared to the group without FI (see Table 2 and Table 3).

Considering the fluoroscopy time, we demonstrated a significant difference between the first 36 procedures of IC 1 without FI (group 1; 18.6 min ± 8.3 min) and his next 36 procedures with FI (group 2; 12.5 min ± 4.5 min) (*p* < 0.001). Comparing the latter group with IC 2 (group 3, with FI) (13.7 min ± 7 min), fluoroscopy time was also significantly shorter (*p* = 0.038). Furthermore, dose area product differed significantly in the first group (IC 1, without FI (5034.1 cGy·cm^2^ ± 4304 cGy·cm^2^)) compared to the second (IC 1 with FI, (4368.6 cGy·cm^2^ ± 2087.3 cGy·cm^2^, *p* = 0.039)) and third group (IC 2, with FI (2787.2 cGy·cm^2^ ± 2284.4 cGy·cm^2^, *p* < 0.001)). The procedure time was significantly longer in the group without FI (group 1, IC 1) with 68.5 min ± 26.8 min significantly longer than in groups 2 and 3 (59 min ± 7.5 min, *p* = 0.004) and group 3 (56.5 min ± 7 min, *p* = 0.003). In the group without FI, 145 mL ± 100 mL contrast medium was used. This was significantly more than in the second group (IC 1, with FI; 63.5 mL ± 30 mL, *p* < 0.001) and in the third group (IC 2, with FI; 55 mL ± 22.5 mL, *p* < 0.001).

### 3.6. Procedural Complications

There was no significant difference in procedural complications comparing group 1 without FI and groups 2 and 3 with FI (see Table 4 and Table 5): 4 patients in the group without FI suffered bleeding with an ISTH/SSC score of 4 (11%). In group 2, there was one patient (*p* = 0.164) whereas in group 3, two patients (*p* = 0.392). Arrhythmias occurred in 3 subjects in the first group only (*p* = 0.077). However, there were no vascular complications in the group without FI. In comparison, there was one vascular complication in the second group and two in the third group (*p* = 0.151). No pericardial effusion, stroke, or dislocation were observed in all groups.

## 4. Discussion

The purpose of this study was to evaluate the impact of real-time FI on the learning curve of interventionalists during left atrial appendage closure.

Based on the analysis of our data, the findings are as follows:

1. The left atrial appendage closure learning curve has a flat course without FI.
2. FI may improve the left atrial appendage closure learning curve.
3. Even highly experienced interventionalists may benefit from FI guidance in their early phase of left atrial appendage closure training.

The initial stages during interventional cardiology training are crucial for acquiring the necessary skills and ensuring patient safety, as the expert consensus summarized the key points of training of new interventionalists: “master the technique, know the device, know the patient” [18,23]. Depending on the complexity of the procedures, the progressions may vary [24]. In contrast to basic diagnostic procedures, such as coronary angiography, more complex interventions, such as structural heart interventions, may require a longer training period [25]. Salemi et al. showed, based on data of 207 interventionalists, that risk-adjusted in-hospital outcomes were improved by increased TAVR experience of operators [26]. Chatriwalla et al. demonstrated that also the operator’s experience of transcatheter mitral valve repair was associated with improvements in procedural success, reduction of procedure time, and procedural complications [27].

**Figure 4:**
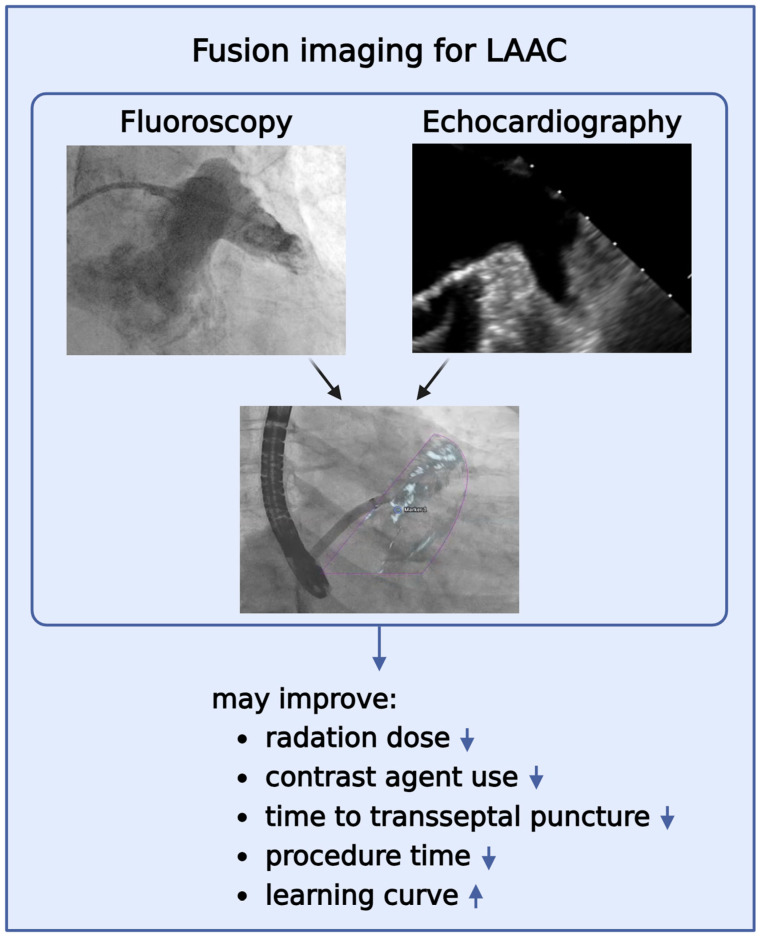
Benefits of FI for LAAC. It may lead to a reduction in radiation dose, contrast agent use, time to transseptal puncture, procedure time, and may optimize the learning curve. Purple triangle depict real-time fluoroscopic and echocardiographic overlay.

Furthermore, structural heart disease interventions require not only enhanced training in technical skills but also thorough training in multimodal imaging in order to gain comprehensive knowledge of cardiac anatomy, particularly for LAAC of the left atrium, LAA, and surrounding structures [28]. Due to the highly variable and complex LAA anatomy, e.g., the LAAC can be challenging [29]. Table 6 and Figure 4 present the current advantages of periprocedural fusion imaging guidance in the field of LAAC. However, the influence on the learning curve has not been yet investigated. Jung et al. demonstrated in a large multicenter study involving 83 hospitals and a total of 13,651 LAAC procedures that 30 cases are necessary to achieve optimal safety of LAAC [12]. Therefore, based on the latter study, we performed our analysis of the learning curves of both interventional cardiologists and divided our study population into three groups with at least >30 patients. Despite the retrospective fashion of our study, we were able to demonstrate that FI guidance leads to a steep improvement in the learning curve already during the initial phase of training compared to the group without FI and a flat learning curve. The pivotal role of imaging during LAAC and its impact on the early operator learning curve was also demonstrated by Wang et al. in a single-center study with 53 patients [30]. Hereby, the application of three-dimensional computed tomographic image guidance to WATCHMAN implantation and the impact on the early operator learning curve was assessed and it demonstrated that it could have a positive impact. In contrast to three-dimensional computed tomographic imagining, FI is a tool that facilitates spatial orientation in real-time, by integrating information from fluoroscopic and echocardiographic images as one overlay in the Cath lab [31]. Furthermore, there were no differences in the occurrence of complications when assessing FI guidance. Interestingly, Ledwoch et al. showed in a study with 90 patients that, the population was divided into three groups, that procedure time, fluoroscopy time, and contrast volume were reduced across the three groups [11]. Furthermore, in-hospital complications decreased significantly and the compression grade of the occluder device was chosen higher with an increasing learning curve. As a matter of fact, our study demonstrated that FI guidance might be useful during training by optimizing the learning curve, thereby achieving less procedure time, decreased amount of contrast agent as well as dose-area product, and less fluoroscopy time, being faster than without FI.

### Limitations

Our study has several limitations, limiting its validity. Our study is a single-center analysis. The data were collected retrospectively. Learning curve analysis was performed on only two interventionalists. Furthermore, the second interventionalist had no experience in LAAC when he started with FI, and FI was not the only aspect of his learning curve, whereas the first interventionist was already experienced and was able to overcome his initial phase of the learning curve without FI. Moreover, the ACP device was initially used for LAAC and switched to Amulet. Based on the current literature, it can be assumed that the switch of devices might have a minor influence on the learning curve of our interventionalists. Further, multicenter, prospective, and randomized studies with more cardiologists (>10) in different training phases and implantations with other LAA closure systems are needed to prove the usefulness of FI during IC training.

## 5. Summary

This is the first study to examine the impact of FI on the learning curve for LAAC. We demonstrated that FI may improve the learning process of interventional cardiologists. FI may be beneficial especially during the first phase of training by facilitating three-dimensional spatial understanding and orientation and thus providing more confidence to an IC in training.

## Figures and Tables

**Table 1:** Patient characteristics.

Variable	Interventionalist 1 −FI, N = 36	Interventionalist 1 +FI, N = 36	Interventionalist 2 +FI, N = 36	*p*-Value
Age (Y), M ± SD	75 ± 9	78 ± 6	75 ± 9	0.222
Height (cm), M ± SD	170.6 ± 8.3	169.8 ± 8.7	171 ± 9.6	0.919
Weight (kg), M ± SD	80.1 ± 14.2	78.6 ± 14.1	80.7 ± 21.9	0.816
BMI (m/kg^2^), M ± SD	27.4 ± 2.2	27.3 ± 4.4	27.5 ± 7.1	0.764
CHA2DS2-VASc-Score	4 ± 1	4 ± 2	4 ± 1	0.919
HAS-BLED-Score	3 ± 1	4 ± 1	2 ± 1	<0.001
Heart failure, N (%)	20 (55.6%)	17 (47.2%)	22 (61.1%)	0.492
CKD, N (%)	19 (52.8%)	17 (47.2%)	13 (36.1%)	0.351
CAD, N (%)	29 (80.6%)	22 (61.1%)	20 (55.6%)	0.064
Heart surgery, N (%)	11 (30.6%)	7 (19.4%)	6 (16.7%)	0.325
PCI, N (%)	20 (55.6%)	14 (38.9%)	11 (30.6%)	0.091
Hypercholesterolemia, N (%)	30 (83.3%)	28 (77.8%)	26 (72.2%)	0.526
Arterial hypertension, N (%)	32 (88.9%)	33 (91.7%)	34 (94.4%)	0.695
Diabetes mellitus, N (%)	10 (27.8%)	12 (33.3%)	12 (33.3%)	0.842
COPD, N (%)	2 (5.6%)	8 (22.2%)	4 (11.1%)	0.100

BMI—Body Mass Index; CKD—Chronic Kidney Disease; CAD—Coronary Artery Disease; PCI—Percutaneous Coronary Intervention; COPD—Chronic Obstructive Pulmonary Disease.

**Table 2:** Comparison of measurements between groups 1 and 2.

Variable	Interventionalist 1 −FI, N = 36	Interventionalist 1 +FI, N = 36	*p*-Value
Fluoroscopy time (min)	18.6 ± 8.3	12.5 ± 4.5	<0.001
Dose area product (cGy·cm^2^)	5034.1 ± 4304	4368.6 ± 2087.3	0.039
Procedure time (min)	68.5 ± 26.8	59 ± 7.5	0.004
Contrast volume (mL)	145 ± 100	63.5 ± 30	<0.001

min—Minutes; mL—Milliliter; cGy—Zentigrey; cm—centimeter; FI—fusion imaging.

**Table 3:** Comparison of measurements between groups 1 and 3.

Variable	Interventionalist 1 −FI, N = 36	Interventionalist 2 +FI, N = 36	*p*-Value
Fluoroscopy time (min)	18.6 ± 8.3	13.7 ± 7	0.038
Dose area product (cGy·cm^2^)	5034.1 ± 4304	2787.2 ± 2284.4	<0.001
Procedure time (min)	68.5 ± 26.8	56.5 ± 7	<0.001
Contrast volume (mL)	163.8 ± 110.5	55 ± 22.5	<0.001

min—Minutes; mL—Milliliter; cGy—Zentigrey; cm—centimeter; FI—fusion.

**Table 4:** Comparison of complications between groups 1 and 2.

Variable	Interventionalist 1 −FI, N = 36	Interventionalist 1 +FI, N = 36	*p*-Value
Pericardial effusion	0	0	-
Bleeding	4 (11%)	1 (3%)	0.164
Vascular complications	0	1 (3%)	0.314
Stroke	0	0	-
Dislocation	0	0	-
Arrhythmia	3 (8%)	0	0.077

FI—fusion imaging.

**Table 5:** Comparison of complications between groups 1 and 3.

Variable	Interventionalist 1 −FI, N = 36	Interventionalist 2 +FI, N = 36	*p*-Value
Pericardial effusion	0	0	-
Bleeding	4 (11%)	2 (5.5%)	0.394
Vascular complications	0	2 (5.5%)	0.151
Stroke	0	0	-
Dislocation	0	0	-
Arrhythmia	3 (8%)	0	0.077

FI—fusion imaging.

**Table 6:** Literature overview on LAAC and fusion imaging.

Authors	Study Design	N (+FI/−FI)	Methods	Results
Afzal et al. [17]	Observational study	155 (34/121)	Echocardiography + fluoroscopy	FI reduced the total procedure time, the time to successful transseptal, and periprocedural amount of contrast agent.
Ebelt et al. [32]	Observational study	75 (25/50)	Echocardiography + fluoroscopy	FI significantly reduced procedure time and the amount of contrast medium
Nelles et al. [33]	Case report	1 (1/0)	Echocardiography + fluoroscopy	FI is safe and feasible
Blusztein et al. [34]	Observational study	31 (31/0)	Echocardiography + fluoroscopy	FI using for zero-contrast LAAC is safe and feasible
Chen et al. [35]	Observational study	82 (41/41)	Computed tomography + fluoroscopy	FI is feasible, safe, and applicable; it reduces the radiation exposure, procedure duration, and volume of contrast media
Roy et al. [36]	Observational study	57 (16/41)	Computed tomography + fluoroscopy	FI reduced contrast volume, procedure time, and fluoroscopy time
Mo et al. [37]	Observational study	117 (39/78)	Computed tomography + fluoroscopy	FI enabled a lower average number of recapture times and the number of devices per patient with a higher one-time successful deployment rate
Peters et al. [38]	Case series	3 (3/0)	Computed tomography + echocardiography	FI improved the detection of LAA anatomy and delivery catheter orientation within the LAA

## Data Availability

The data presented in this study are available on request from the corresponding author.

## Supplementary Materials

The following supporting information can be downloaded at: https://www.mdpi.com/article/10.3390/jcdd11030082/s1, Videos S1–S5: we have depicted 5 cases demontrating FI during LAAC, not only facilitating spatial orientation in real-time but also integrating information from fluoroscopic and echocardiographic images as one overlay.

## Abbreviations

BMI	Body Mass Index
CAD	Coronary Artery Disease
CKD	Chronic Kidney Disease
COPD	Chronic Obstructive Pulmonary Disease
CV	Contrast Volume
DAP	Dose Area Product
EAPCI	European Association of Percutaneous Cardiovascular Interventions
FI	Fusion Imaging
FT	Fluoroscopy Time
IC	Interventional Cardiologist
IQR	Interquartile Range
LA	Left Atrium
LAA	Left Atrial Appendage
LAAC	Left Atrial Appendage Closure
MACE	Major Adverse Cardiac Events
NOAC	Novel oral anticoagulant
PT	Procedure Time
RAO	Right Anterior Oblique
SD	Standard Deviation
TEE	Transoesophageal Echocardiography
TSP	Transseptal Puncture
VKA	Vitamin K Antagonist
